# Three-Dimensional Breast Radiotherapy and the Elective Radiation Dose at the Sentinel Lymph Node Site in Breast Cancer

**DOI:** 10.1245/s10434-015-4413-7

**Published:** 2015-02-24

**Authors:** Lori M. van Roozendaal, Robert-Jan Schipper, Leonie H. M. Smit, Boudewijn T. Brans, Regina G. H. Beets-Tan, Marc B. I. Lobbes, Liesbeth J. Boersma, Marjolein L. Smidt

**Affiliations:** 1 Department of Surgical Oncology, Maastricht University Medical Center, Maastricht, The Netherlands; 2 Department of Radiology and Nuclear Medicine, Maastricht University Medical Center, Maastricht, The Netherlands; 3 Department of Radiation Oncology, Maastricht University Medical Center (MAASTRO Clinic), Maastricht, The Netherlands; 4 GROW-School for Oncology and Developmental Biology, Maastricht University Medical Center, Maastricht, The Netherlands

## Abstract

**Background:**

Several trials are presently randomizing clinically node-negative breast cancer patients treated with breast-conserving therapy (BCT) to sentinel lymph node biopsy (SLNB) or watchful waiting. We aimed to investigate the elective radiation dose at the sentinel lymph node (SLN) site while evaluating two techniques for SLN localization, in breast cancer patients treated with lumpectomy and three-dimensional (3D) whole-breast radiotherapy.

**Methods:**

The SLN site of consecutive Tis-2N0 breast cancer patients undergoing lumpectomy and forward intensity-modulated whole-breast radiotherapy was determined by the location of the hotspot on preoperative single-photon emission computed tomography (SPECT)/computed tomography (CT) and by a surgical clip placed at the removed SLN(s) during SLNB. The radiation dose at the SLN site was subsequently determined on the postoperative radiotherapy planning CT. An elective radiation dose to the SLN site was defined as at least 95 % of the breast dose.

**Results:**

Of the 42 included patients, the mean percentage of the breast dose on the SLN site was 90 % (standard deviation 26, range 7–132, median 99), with a non-significant difference between the two techniques (surgical clip or SPECT/CT) (*p* = 0.608). In 32/42 patients (76 %) the SLN site received an elective radiation dose.

**Conclusions:**

A surgical clip placed at the removed SLN(s) during SLNB proved to be an adequate method of determining the radiation dose at the SLN site when compared with using SPECT/CT. With the use of 3D radiotherapy, the site of the SLN is treated with an elective radiation dose in the majority of patients who are treated with BCT.

The treatment of breast cancer with breast-conserving therapy (BCT) as a less invasive alternative to mastectomy was introduced in the early 1980s. The addition of whole-breast radiotherapy to lumpectomy resulted in not only lower local recurrence rates but also lower regional recurrence rates.^1^,^2^ A meta-analysis showed that in sentinel lymph node (SLN)-negative patients, whole-breast radiotherapy after lumpectomy is associated with a lower risk of axillary recurrence (odds ratio 0.29; *p* < 0.001).^3^ In the American College of Surgeons Oncology Group (ACOSOG) Z0011 trial, SLN-positive patients treated with BCT were randomized to completion axillary lymph node dissection or watchful waiting.^4^ The regional recurrence rate was not increased in the watchful-waiting arm, while it is estimated that 27 % had residual nodal disease that was not surgically removed.^5^ It is suggested that these findings might be partly explained by incidental irradiation of the lower axilla by whole-breast radiotherapy.^6^,^7^ Studies have shown that up to 73 % of axillary level I and 26 % of level II receive an elective radiation dose with conventional two-dimensional (2D) whole-breast radiotherapy.^8^−^10^

Presently, several independent trials are randomizing clinically node-negative breast cancer patients treated with BCT to SLN biopsy (SLNB) or watchful waiting—the Dutch BOOG 2013-08, Italian SOUND, and British SNIPE trials.^11^−^13^ Studies have shown that the SLN site is radiated in 78–94 % of patients using conventional 2D whole-breast radiotherapy.^14^−^16^ Meanwhile, three-dimensional (3D) radiotherapy techniques mostly replaced 2D radiotherapy. Both 3D-conformal radiotherapy (3D-CRT) and (forward) intensity-modulated radiotherapy (IMRT) are considered 3D radiotherapy techniques. In 3D-CRT, the shape of the radiation field is adapted to the shape of the target volume but can still lead to high doses in non-target volumes. In IMRT, the shape of the high-dose volume is as conformal as possible to the 3D shape of the target volume, and, furthermore, delivers a more homogeneous dose to the target volume than 3D-CRT.^10^ IMRT can thus be considered as a more advanced and more accurate type of 3D radiotherapy. It is questionable whether, with a contemporary radiation technique, the SLN still receives an elective radiation dose as more advanced radiotherapy results in more accurate irradiation of the targeted volume and less dose to non-target volumes.

Previous studies on the radiation dose at the SLN site used surgical clips as a marker, representing the postoperative situation of a minimally dissected axilla. As trials are investigating the safety of omitting the SLNB, we were interested in the SLN site in the undissected axilla. For this purpose, preoperative single-photon emission computed tomography (SPECT)/computed tomography (CT) images seem more appropriate and were therefore performed in a proportion of patients.

We aimed to investigate whether the radiation dose at the surgical clip that is placed at the location of the removed SLN(s) during SLNB corresponds to the dose at the region of increased tracer uptake (the hotspot) on SPECT/CT, and, furthermore, whether the SLN site receives an elective radiation dose, in breast cancer patients treated with lumpectomy, SLNB, and whole-breast radiotherapy using forward IMRT.

## Methods

### Study Population

Consecutive patients diagnosed with unifocal cTis-2N0 breast cancer or carcinoma in situ of the breast between March 2013 and January 2014, and were about to undergo BCT and SLNB in our breast center, were included. Patients receiving completion axillary treatment or with a history of surgery or radiotherapy of the ipsilateral breast or axilla were excluded. The acquisition of informed consent was waived by the Medical Ethics Committee (MEC UM 13-4-019). One group of patients (*n* = 10) had both preoperative SPECT/CT images of the axillary region and a surgical clip placed at the location of the removed SLNs to evaluate if these methods were equivalent, while a second group of patients (*n* = 32) had only the surgical clip placed at the SLN site.

### Sentinel Lymph Node Biopsy

For the SLNB, a total of 80 MBq Technetium-99 nanocolloid (Nanocoll^®^; GE Healthcare, Eindhoven, The Netherlands) was injected intradermally in the periareolar region, followed by lymphoscintigraphic images. Additionally, integrated SPECT/CT images were available in patients who participated in an ongoing prospective study concerning non-invasive nodal staging (MEC UM 12-0-36). All SPECT/CT images were performed using the same positioning and fixation system as used during the radiotherapy planning CT and treatment, with the patient in the supine position, the head in a contoured headrest, and both arms above the head in the cranial arm holder combined with two upper-arm supports (Posirest™-2 system; Cablon Medical B.V., Leusden, The Netherlands). Two hours after injection, the SPECT/CT images were obtained (Precedence SPECT/6-slice CT scanner; Philips, Best, The Netherlands).

The SLN(s) were identified during surgery by using the lymphoscintigraphic images, blue dye (Bleu Patente V; Guerbet, Aulnay-sous-Bois, France) and gamma probe. The surgeon placed a surgical clip(s) at the location of the removed SLN(s).

### Radiotherapy Planning Scans

All patients were routinely scheduled for a radiotherapy planning CT within 5 weeks after surgery (SOMATOM Sensation 10; Siemens, Forchheim, Germany). Patients were positioned as described in the previous section.

### Radiation Therapy Details

The prescribed breast dose was 16 × 2.67 Gy in 23 patients; 16 patients received a modified scheme with a low boost (21 × 2.17 Gy and simultaneous integrated boost [SIB] of 21 × 2.66 Gy); and three patients received a modified scheme with a high boost (23 × 2.03 Gy and SIB of 23 × 2.66 Gy). Delineation of the breast and tumor bed was performed as described earlier.^17^,^18^ In short, for delineation of the breast, a radiopaque wire was used to mark the palpable breast tissue. The clinical target volume (CTV) of the breast was defined 5 mm under the skin surface, in front of the major pectoral muscle and chest wall, borders largely determined by the radiopaque wire. Cranially, the CTV breast did not extend above the superior border of the sternoclavicular joint, and the medial border of the CTV did not extend beyond the lateral edge of the sternum. The tumor bed was delineated using all preoperative available information, such as mammography, magnetic resonance imaging (MRI) and physical examination. For the CTV of the tumor bed, a margin of 1.5 cm minus the minimally free resection margin was applied around the tumor bed. The CTV of the tumor bed did not extend into the chestwall, and remained 5 mm beneath the skin. CTV planning target volume (PTV) expansion was 5 mm in all directions for both the breast and the tumor bed, except towards the skin surface where no CTV–PTV margin was taken.

The forward IMRT technique was used for treatment planning, as described earlier.^18^ With this technique we always start with two open tangential beams, and we optimize the dose distribution with additional segments and/or beams. In our patient population we found that additional segments (<3 segments) were needed in one-third of patients, and additional beams (<2 beams) were needed in two-thirds of patients.

The dose homogeneity constraints for the PTVs were kept to vary between 95 and 107 % of the prescribed dose. The lung constraints consisted of a central lung distance below 3 cm, the mean lung dose had to be <7.5 Gy, the heart constraints consisted of a maximum heart distance below 1 cm, the heart volume receiving >10 Gy had to be <5 %, and the heart volume receiving >5 Gy had to be <10 %.^19^ Moderately voluntary breath-holding techniques to reduce heart dose in left-sided breast cancer were applied.^18^

The radiation oncologists and radiation technicians were blinded for the study aim and procedure at the time of treatment of all patients.

### Data Collection and Statistical Analyses

Data concerning diagnosis and treatment were collected. The *x*, *y*, and *z* axis coordinates of the center of the SPECT hotspot were determined on SPECT/CT, after which they were converted by using the *x*, *y*, and *z* axis coordinates (0,0,0) of the planning CT scan, followed by visualization of these coordinates on the planning CT scan. The surgical clip was also visualized on the planning CT scan. The corresponding radiation dose, expressed as a percentage of the prescribed breast dose, was determined at the location of the SPECT hotspot and the surgical clip, at the center of these points in both techniques. In case of multiple clips due to the removal of several SLNs, the lowest radiation dose was obtained. An elective radiation dose to the SLN site was defined as at least 95 % of the prescribed breast dose.

Descriptive categorical data are presented as proportions and absolute numbers. Continuous variables are presented as means with standard deviations (SD). The Bland–Altman and one-sample *t*-tests were used to analyze the agreement between the radiation dose delivered to the surgical clip and the hotspot, with 95 % limits of agreement. Fisher’s exact test was used to analyze if the location of the tumor in the breast, the size of the breast, or a left- versus right-sided lesion were predictors for not receiving an elective radiation dose at the SLN site, and binary logistic regression for body mass index (BMI), radiation therapy boost, and a tumor located in the laterocranial quadrant. Statistical analyses were performed using Statistical Package for the Social Sciences (SPSS), version 20.0 (IBM Corporation, Armonk, NY, USA). A *p* value of <0.05 was considered statistically significant.

## Results

Mean age of the ten patients with both SPECT/CT images and a surgical clip was 63 years (SD 11, range 50–79, median 63) and mean tumor size was 12 mm (SD 8, range 4–26, median 11). Radiation dose on the SPECT hotspot and surgical clip of the ten patients are presented in Table 1. Mean radiation dose on the SPECT hotspot was 38.92 Gy (SD 8, range 20–45) and on the surgical clip 38.62 Gy (SD 9, range 16–45). The mean percentage of the breast dose at the SPECT hotspot and the surgical clip was 89 % (SD 17, range 48–105) and 88 % (SD 20, range 37–104), respectively. The mean difference between the two methods was −0.0076 (95 % limits of agreement ±0.0899) (*p* = 0.608).

**Table 1:** Radiation dose and percentage of prescribed whole-breast dose on SPECT hotspot and surgical clip in ten patients

	Radiation scheme to the whole breast	Total prescribed dose to breast (Gy)	SPECT hotspot		Surgical clip	
			Dose (Gy)	Percentage of total breast dose (%)	Dose (Gy)	Percentage of total breast dose (%)
1	16 × 2.67	42.72	35.50	83.10	37.20	87.08
2	16 × 2.67	42.72	44.60	104.40	44.58	104.35
3	16 × 2.67	42.72	32.40	75.84	30.88	72.28
4	16 × 2.67	42.72	41.50	97.14	41.10	96.21
5	16 × 2.67	42.72	20.40	47.75	15.90	37.22
6	16 × 2.67	42.72	41.50	97.14	41.65	97.50
7	21 × 2.17	45.57	42.00	92.17	43.40	95.24
8	21 × 2.17	45.57	42.80	93.92	43.46	95.37
9	21 × 2.17	45.57	43.50	95.46	45.10	98.97
10	16 × 2.67	42.72	44.97	105.27	42.90	100.42

*Radiation scheme*: the number of *fx* × dose per *fx* in Gy to the whole breast

*SPECT* single-photon emission computed tomography

**Fig. 1:**
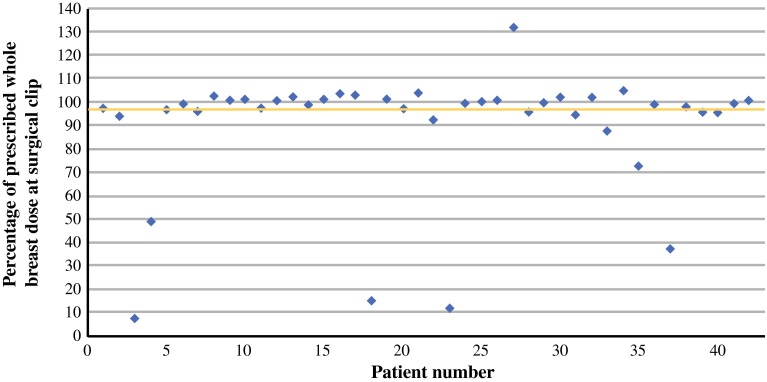
Percentage of prescribed whole-breast dose on the surgical clip at the SLN site in 42 patients. The *yellow line* represents 95 % of the prescribed whole-breast dose at the surgical clip. *SLN *sentinel lymph node

**Fig. 2:**
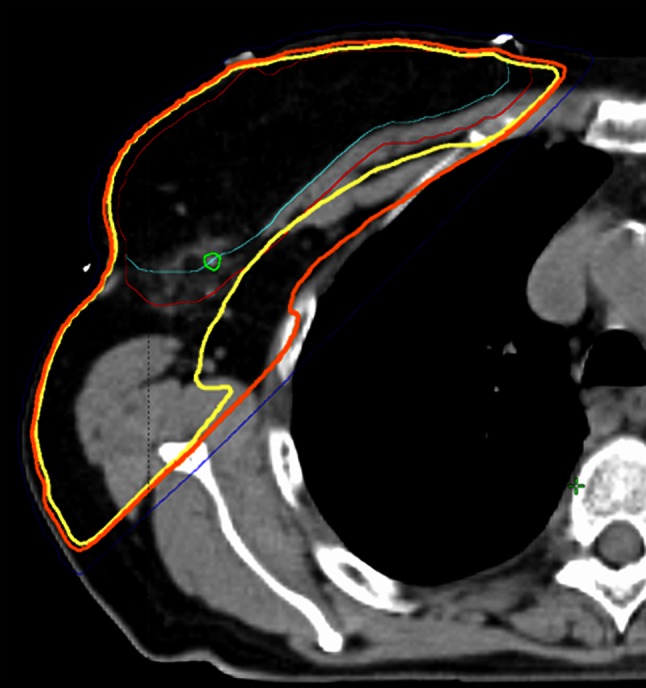
Radiotherapy planning CT of a study patient after lumpectomy and SLNB with an elective radiation dose at the surgical clip. The breast tumor of this patient was located in the mediocaudal quadrant of the breast; the tumor bed is not visible on this CT image. The *yellow line* contouring the breast represents the 95 % isodose, i.e. all spots on this line received at least 95 % of the prescribed dose; the *orange line* represents the 90 % isodose; the *dark blue line* represents the 50 % isodose; the *light blue line* represents the clinical target volume of the breast; and the *red line* represents the planning target volume of the breast. The surgical clip at the SLN site is contoured by a *green line*. *CT* computed tomography, *SLNB *sentinel lymph node biopsy, *SLN* sentinel lymph node

Because the percentages of the elective radiation dose at the surgical clip and the SPECT hotspot were in line, further analyses were performed on all 42 patients. Patient demographics and tumor characteristics of the 42 patients are shown in Table 2. The percentages of the breast dose on the surgical clip of all patients are shown in Fig. 1. The mean percentage of the breast dose on the surgical clip was 90 % (SD 26, range 7–132, median 99 %). The SLN site received at least an elective radiation dose in 32 patients (76 %). An example of a patient with an elective radiation dose on the surgical clip is shown in Fig. 2. In 35 of 42 patients (83 %), at least 90 % of the breast dose was delivered, at least 80 % in 36 patients (86 %), and at least 50 % in 37 patients (88 %) (Table 3). The location of the tumor in the breast, the size of the breast, a left- versus right-sided lesion or BMI, and the combination of radiation therapy boost with a tumor located in the laterocranial quadrant did not influence the elective radiation dose at the SLN site.

**Table 2:** Patient demographics and tumor characteristics of all patients

Characteristic	Value
Number of patients	42
Age, years	
Mean (SD)	66 (9)
Range	48–81
Tumor size, mm	
Mean (SD)	17 (10)
Range	4–50
Number of sentinel lymph nodes removed	
Mean (SD)	1.45 (0.8)
Range	1–4
Number of non-sentinel lymph nodes removed	
Mean (SD)	0.5 (0.8)
Range	0–3
pT-stadium [*N* (%)]	
Tis	3 (7)
T1	29 (69)
T2	10 (24)
pN-stadium [*N* (%)]	
pN0	36 (86)
pN0(i +)	1 (2)
pN1mi	4 (10)
pN1	1 (2)
Tumor type [*N* (%)]	
Ductal carcinoma in situ	3 (7)
Invasive ductal carcinoma, NOS	32 (76)
Invasive lobular carcinoma, NOS	3 (7)
Other	4 (10)
Affected side [*N* (%)]	
Left	21 (50)
Right	21 (50)
Breast cup size [*N* (%)]	
A	2 (5)
B	12 (29)
C	13 (31)
D	5 (12)
E	3 (7)
F	2 (5)
G	2 (5)
Unknown	3 (7)
Location of tumor in breast [*N* (%)]	
Laterocranial quadrant	23 (55)
Laterocaudal quadrant	7 (17)
Mediocranial quadrant	5 (12)
Mediocaudal quadrant	7 (17)
BMI score [*N* (%)]	
18.5–25	17 (41)
25–30	17 (41)
30–35	6 (14)
> 35	2 (5)

*SD* standard deviation, *NOS* not otherwise specified, *BMI* body mass index

**Table 3:** Number of patients receiving at least 95, 90, 85, 80 or 50 % of the prescribed whole-breast dose on surgical clip

Percentage of prescribed whole-breast dose	Number of patients	Percentage of patients
≥95	32	76
≥90	35	83
≥85	36	86
≥80	36	86
≥50	37	88

## Discussion

This study aimed to investigate the incidental radiation dose to the SLN site in breast cancer patients treated with lumpectomy followed by whole-breast radiotherapy using forward IMRT, to illustrate incidental treatment of the SLN in trials investigating the safety of omitting the SLNB. A surgical clip placed at the location of the removed SLN(s) during SLNB proved to be an adequate method to determine the radiation dose at the SLN site when compared with using SPECT/CT images. An elective radiation dose, i.e. >95 % of the total breast dose, was delivered to the SLN site in 76 % of patients.

The hotspot on SPECT/CT represents the preoperative localization of the SLN site in an undissected axilla. This method therefore seems appropriate to simulate the situation where the SLNB is omitted. The SLN site can be visualized without postoperative effects, such as hematoma or seroma, which can influence the configuration of the lymph nodes within the axilla. However, there are some limitations in the use of SPECT/CT for accurate localization of the SLN site. At first, the attenuation of the SPECT and CT images can be technically challenging, for example due to respiratory motion artifacts.^20^ Second, the composition of the axilla could change by (oncoplastic) surgery of the breast. In that case, the surgical clip method might be more appropriate for adequate SLN localization. However, results of our study showed that the percentage of the prescribed breast dose at the hotspot was in line with the surgical clip in ten patients.

3D-CRT and forward IMRT have now mostly replaced conventional 2D radiotherapy for breast irradiation. The benefit of 3D radiotherapy includes more accurate irradiation of the targeted volume and reduced toxicity to surrounding tissue. In whole-breast radiotherapy this might result in a decreased incidental radiation dose to, among others, the axilla, with the hypothetical advantage of a decreased risk of lymphedema and fibrosis.^21^ On the contrary, a reduced dose to the axilla could negatively affect the incidental benefit of a decreased regional recurrence rate in patients treated with BCT.^3^ As conventional 2D radiotherapy instead of 3D radiotherapy techniques were routinely used in the ACOSOG Z0011 trial, it is suggested that radiotherapy should be extended to include axillary level I–II to maintain excellent regional control when applying Z0011 results in today’s practice.^22^ Indeed, Kataria et al. showed a relatively small, although significant, difference in the axillary radiation dose when comparing conventional 2D radiotherapy with 3D-CRT or IMRT. The mean percentage of axillary level I that received at least 90 % of the total breast dose was 73 % for conventional 2D radiotherapy, 57 % (*p* = 0.029) for 3D-CRT, and 49 % (*p* = 0.029) for IMRT; for level II, the mean percentage was 25, 41 % (*p* = 0.028), and 35 % (*p* = 0.068), respectively.^10^ The current study was the first to investigate the radiation dose to the SLN site with 3D breast radiotherapy, and, more specifically, with IMRT. The elective radiation dose to the SLN in 76 % of patients in our study is in line with the percentage of 78 % reported by Rabinovitch et al., and lower than the 85 % reported by Schlembach et al. and the 94 % reported by Chung et al., which were all studies using conventional 2D breast radiotherapy.^14^−^16^ As described earlier, IMRT can be considered as a more advanced and more accurate type of 3D radiotherapy when compared with 3D-CRT. Together with results of the study of Kataria et al.,^10^ it is likely that a higher percentage of patients will have an elective radiation dose to the SLN when evaluating 3D-CRT. Therefore, we find that even with contemporarary 3D techniques, the SLN still receives an adequate dose in the majority of patients.

The location of the tumor in the breast, the size of the breast, a left- versus right-sided lesion, a radiation therapy boost, and BMI are sometimes referred to as factors influencing the amount of radiation to the axilla. Our study failed to demonstrate a relation between these factors and not receiving an elective radiation dose at the SLN site. The location of the SLN posteriorly or high in the axilla is more likely to explain why these patients have a lower radiation dose at the SLN, but we did not analyze this in our cohort. The study by Uren et al. showed that the axillary SLN is located anteriorly or centrally in axillary level I in 83 % of patients, and the remaining proportion more posteriorly in level I or in level II–III.^23^ Furthermore, the site of the tracer injection for SLNB influences the drainage pattern and thus the location where the SLN appears. In periareolar injections, as performed in our cohort, a higher proportion of patients have drainage to the anterior region of axillary level I (57 %) compared with peritumoral injections (46 %) (*p* = 0.042).^23^

In the current study, an elective radiation dose was delivered to the SLN site in 76 % of patients, and in 86 % of patients the SLN site received at least 85 % of the elective radiation dose (Table 3). The optimal dose for curative radiation of metastatic disease in lymph nodes is questionable and might be less than 95 % of the prescribed breast dose. Lower doses could result in a decreased effect but might still be effective in subclinical disease. Evidence exists for sufficient control of several cancer types with low radiation doses ranging from 10 to 30 Gy.^24^,^25^ Withers and Suwinski proposed that the dose response curve for microscopic disease is different from the curve for macroscopic disease, and that a lower radiation dose in these patients is probably equally effective.^26^ However, because the amount of residual subclinical disease differs among patients, it is not possible to predefine the elective radiation dose for the individual patient.

For the comparison of SPECT/CT and the surgical clip as methods for determining the elective radiation dose at the SLN site, the data of ten patients were used in our study. It is not expected that results would be affected if more patients had been analyzed because the percentage of patients receiving an elective radiation dose at the SLN site is comparable to what is shown in the available literature.^14^−^16^ Final analyses in this study were performed on existing radiation therapy plans of 42 patients, and we did not assess delineation and inter-observer variation, but the radiation oncologists and radiation technicians were blinded for the study aim and procedure at the time of treatment of all patients. Despite the growing popularity of partial breast radiotherapy, we did not include patients receiving this treatment in our analysis because in The Netherlands it is almost only applied within clinical trials and because the mentioned trials investigating the safety of omitting the SLNB only include clinically node-negative breast cancer patients when they are treated with lumpectomy and whole-breast radiotherapy.^11^−^13^ It can be expected that for partial breast radiotherapy, the amount of radiation to the SLN is highly influenced by the location of the tumor in the breast, while this was not the case in our cohort.

## Conclusions

A surgical clip placed at the location of the removed SLN during SLNB proved to be an adequate method of determining the radiation dose at the SLN site when compared with using SPECT/CT images. With the use of 3D radiotherapy, the site of the SLN is treated with an elective radiation dose in the majority of patients who are treated with BCT. Whole-breast radiotherapy after lumpectomy in patients in whom the SLNB is omitted might therefore help to prevent an increase of the regional recurrence rate. The safety of omitting the SLNB should be confirmed by the initiated randomized controlled trials.

## References

[1] Fisher B, Anderson S, Bryant J (2002). Twenty-year follow-up of a randomized trial comparing total mastectomy, lumpectomy, and lumpectomy plus irradiation for the treatment of invasive breast cancer. N Engl J Med..

[2] Early Breast Cancer Trialists’ Collaborative Group, Darby S, McGale P, et al (2011). Effect of radiotherapy after breast-conserving surgery on 10-year recurrence and 15-year breast cancer death: meta-analysis of individual patient data for 10,801 women in 17 randomised trials. Lancet..

[3] van Wely BJ, Teerenstra S, Schinagl DA, Aufenacker TJ, de Wilt JH, Strobbe LJ (2011). Systematic review of the effect of external beam radiation therapy to the breast on axillary recurrence after negative sentinel lymph node biopsy. Br J Surg..

[4] Giuliano AE, Hunt KK, Ballman KV (2011). Axillary dissection vs no axillary dissection in women with invasive breast cancer and sentinel node metastasis: a randomized clinical trial. JAMA..

[5] Giuliano AE, McCall L, Beitsch P (2010). Locoregional recurrence after sentinel lymph node dissection with or without axillary dissection in patients with sentinel lymph node metastases: the American College of Surgeons Oncology Group Z0011 randomized trial. Ann Surg..

[6] Alco G, Dincer M (2013). Are the standard tangential breast irradiation fields used in the ACOSOG Z0011 trial really covering the entire axilla?. Ann Surg..

[7] Belkacemi Y, Allab-Pan Q, Bigorie V (2013). The standard tangential fields used for breast irradiation do not allow optimal coverage and dose distribution in axillary levels I–II and the sentinel node area. Ann Oncol..

[8] Reznik J, Cicchetti MG, Degaspe B, Fitzgerald TJ (2005). Analysis of axillary coverage during tangential radiation therapy to the breast. Int J Radiat Oncol Biol Phys..

[9] Reed DR, Lindsley SK, Mann GN (2005). Axillary lymph node dose with tangential breast irradiation. Int J Radiat Oncol Biol Phys..

[10] Kataria T, Bisht SS, Gupta D (2013). Incidental radiation to axilla in early breast cancer treated with intensity modulated tangents and comparison with conventional and 3D conformal tangents. Breast..

[11] van Roozendaal LM, de Wilt JHW, Smidt ML. Clinically node negative breast cancer patients undergoing breast conserving therapy: follow-up versus sentinel lymph node biopsy. 35th Annual San Antonio Breast Cancer Symposium; 4–8 Dec 2012: San Antonio (TX).

[12] Gentilini O, Veronesi U (2012). Abandoning sentinel lymph node biopsy in early breast cancer? A new trial in progress at the European Institute of Oncology of Milan (SOUND: Sentinel node vs Observation after axillary UltraSouND). Breast..

[13] Nadeem RM (2013). The feasibility of SNIPE trial; sentinel lymph node biopsy vs. no-SLNB in patients with early breast cancer. Cancer Res..

[14] Rabinovitch R, Ballonoff A, Newman F, Finlayson C (2008). Evaluation of breast sentinel lymph node coverage by standard radiation therapy fields. Int J Radiat Oncol Biol Phys..

[15] Schlembach PJ, Buchholz TA, Ross MI (2001). Relationship of sentinel and axillary level I–II lymph nodes to tangential fields used in breast irradiation. Int J Radiat Oncol Biol Phys..

[16] Chung MA, DiPetrillo T, Hernandez S, Masko G, Wazer D, Cady B (2002). Treatment of the axilla by tangential breast radiotherapy in women with invasive breast cancer. Am J Surg..

[17] Boersma LJ, Janssen T, Elkhuizen PH (2012). Reducing interobserver variation of boost-CTV delineation in breast conserving radiation therapy using a pre-operative CT and delineation guidelines. Radiother Oncol..

[18] Peulen H, Hanbeukers B, Boersma L (2012). Forward intensity-modulated radiotherapy planning in breast cancer to improve dose homogeneity: feasibility of class solutions. Int J Radiat Oncol Biol Phys..

[19] Hurkmans CW, Borger JH, Bos LJ (2000). Cardiac and lung complication probabilities after breast cancer irradiation. Radiother Oncol..

[20] Seo Y, Mari C, Hasegawa BH (2008). Technological development and advances in single-photon emission computed tomography/computed tomography. Semin Nucl Med..

[21] Bentzen SM, Dische S (2000). Morbidity related to axillary irradiation in the treatment of breast cancer. Acta Oncol..

[22] Haffty BG, Hunt KK, Harris JR, Buchholz TA (2011). Positive sentinel nodes without axillary dissection: implications for the radiation oncologist. J Clin Oncol..

[23] Uren RF, Howman-Giles R, Chung DK (2012). SPECT/CT scans allow precise anatomical location of sentinel lymph nodes in breast cancer and redefine lymphatic drainage from the breast to the axilla. Breast..

[24] Hatfield P, Cooper R, Sebag-Montefiore D (2008). Involved-field, low-dose chemoradiotherapy for early-stage anal carcinoma. Int J Radiat Oncol Biol Phys..

[25] Marks LB (1990). A standard dose of radiation for “microscopic disease” is not appropriate. Cancer..

[26] Withers HR, Suwinski R (1998). Radiation dose response for subclinical metastases. Semin Radiat Oncol..

